# Use of milk proteins as biomarkers of changes in the rumen metaproteome of Holstein cows fed low-fiber, high-starch diets

**DOI:** 10.3168/jds.2022-22910

**Published:** 2023-05-18

**Authors:** B. K. Mulakala, K. M. Smith, M. A. Snider, A. Ayers, M. C. Honan, S. L. Greenwood

**Affiliations:** 1 Department of Animal and Veterinary Sciences, University of Vermont, Burlington, VT 05405; 2 William H. Miner Agricultural Research Institute, Chazy, NY 12921; 3 Department of Agriculture, Southeast Missouri State University, Cape Girardeau, MO 63701; 4 Department of Animal Science, University of California, Davis, Davis, CA 95616

**Keywords:** rumen-fermentable starch, physically effective undegraded neutral detergent fiber, rumen microbial proteins, milk proteins

## Abstract

Dietary levels of undegraded neutral detergent fiber (uNDF240) and rumen-fermentable starch (RFS) can affect the rumen microbiome and milk composition. The objective of the study is to investigate the use of milk proteins as biomarkers of rumen microbial activity through a comparative evaluation of the rumen microbial and milk protein profiles produced by Holstein cows fed diets with varying contents of physically effective uNDF240 (peuNDF240) and RFS. Eight ruminally cannulated lactating Holstein cows were included in a larger study as part of a 4 × 4 Latin square design with 4 28-d periods to assess 4 diets varying in peuNDF240 and RFS content. For this experiment, cows received one of 2 dietary treatments: (1) low-peuNDF240, high-RFS (LNHR) diet or (2) high-peuNDF240, low-RFS (HNLR) diet. Within each period, rumen fluid samples were collected from each cow on d 26 (1400 h) and d 27 (0600 h and 1000 h), and milk samples were collected from each cow on d 25 (2030 h), d 26 (0430 h, 1230 h, and 2030 h), and d 27 (0430 h and 1230 h). Microbial proteins were isolated from each rumen fluid sample. For milk samples, milk proteins were fractionated, and the whey fraction was subsequently isolated. Isolated proteins within each rumen fluid or milk sample were isobarically labeled and analyzed by liquid chromatography-tandem mass spectrometry. Product ion spectra acquired from rumen fluid samples were searched using SEQUEST against 71 composite databases. In contrast, product ion spectra acquired from milk samples were searched against the *Bos taurus* database. Data were analyzed using the PROC MIXED procedure in SAS 9.4 to assess the effect of diet and time of sampling. To increase stringency, the false discovery rate-adjusted *P*-value (*P*_FDR_) was also calculated to account for multiple comparisons. Using the mixed procedure, a total of 129 rumen microbial proteins were quantified across 24 searched microbial species. Of these, the abundance of 14 proteins across 9 microbial species was affected due to diet and diet × time interaction, including 7 proteins associated with energetics pathways. Among the 159 quantified milk proteins, the abundance of 21 proteins was affected due to the diet and diet × time interaction. The abundance of 19 of these milk proteins was affected due to diet × time interactions. Of these, 16 proteins had the disparity across diets at the 0430 h sampling time, including proteins involved in host defense, nutrient synthesis, and transportation, suggesting that biological shifts resulting from diet-induced rumen changes are not diurnally uniform across milkings. The concentration of lipoprotein lipase (LPL) was statistically higher in the milk from the cows fed with the LNHR diet, which was numerically confirmed with an ELISA. Further, as determined by ELISA, the LPL concentration was significantly higher in the milk from the cows fed with the LNHR diet at 0430 h sampling point, suggesting that LPL concentration may indicate dietary carbohydrate-induced ruminal changes. The results of this study suggest that diet-induced rumen changes can be reflected in milk in a diurnal pattern, further highlighting the need to consider sampling time points for using milk proteins as a representative biomarker of rumen microbial activity.

## INTRODUCTION

Diets formulated for high-producing dairy cows typically include a high proportion of rapidly fermentable carbohydrates to meet the cow’s energy demands. However, feeding dairy cows rich in rapidly fermentable carbohydrates and low in fiber can affect their DMI, performance, and milk quality (Grant, 1997; Zebeli et al., 2008; Fustini et al., 2017). For example, Akhlaghi et al. (2022) reported that cows fed a high-starch, low-fiber diet (29.2% of DM, 32.9% DM) produced, on average, 1.2 kg/d more milk, and consumed an additional 0.04 kg DM/d, compared with cows fed a low-starch, high-fiber diet (22.3% of DM, 29.2% of DM). To maintain healthy ruminal function, the NASEM (2021) recommends a minimum of 25 to 33% fiber (on a DM basis) for lactating cows, measured as NDF content. In addition to the fiber content of the diet, its physical characteristics such as particle size, digestibility, effect of ruminal fill, and daily DMI (Conrad et al., 1964). To better characterize the fibrous content of the diet, Mertens (1997) proposed the concept of physically effective NDF (**peNDF**), which is calculated by multiplying the NDF content by its physical effectiveness factor (**pef**). The estimation of pef equates to the proportion of particles retained on the ≥4 mm sieve of the Penn State Particle Separator (Heinrichs and Kononoff, 2013). A common method for measuring the digestibility of fiber is the determination of undegraded NDF at 240 h (**uNDF240**). The uNDF240 is the percentage of NDF that is not degraded after 240 h of incubation with mixed ruminal microbes (Allen and Mertens, 1988, Raffrenato et al., 2018). Recently, Grant et al. (2020) proposed the concept of physically effective undegraded NDF at 240 h (**peuNDF240**), which summarizes the chemical and physical properties of fiber; it is calculated by multiplying the uNDF240 by the pef. Previously, Smith (2021) reported that diets containing lower peuNDF240 (~6% of DM) and higher rumen-fermetable starch (**RFS**; 19% of DM) decreased milk fat %, total-tract digestibility of NDF, and acetate:propionate ratio in dairy cows. Therefore, an optimal balance between peuNDF240 fractions and RFS is important to optimizing nutrient utilization and productivity.

The effect of dietary carbohydrate profile on animal productivity is mainly the result of a modified ruminal microbial environment, and several studies have reported a shifting rumen microbiota during feeding of diets containing a high proportion of rapidly fermentable carbohydrates with inadequate fiber content (Hook et al., 2011; Ogunade et al., 2019; Ramos et al., 2021). Methodologically, these studies investigated rumen microbial changes at the DNA (meta-genomics) and RNA (meta-transcriptomics) levels; however, these studies did not assess the actual metabolic response of the microbial population at the protein level. Previously, Hart et al. (2018) reported the mismatch between the cows’ rumen microbial transcriptomic and translated data. More recently, Mulakala et al. (2022) characterized the rumen metaproteome affected by varying levels of peuNDF240 and RFS content in the diet fed to lactating dairy cows.

The effect of diet-induced modifications to the rumen microbial environment alters the cow’s metabolic status, which is commonly reflected by alterations in body fluids such as blood, urine, saliva, and milk (Overton et al., 2017). The standard method used for diagnosing metabolic diseases is the analysis of key factors in blood circulation (Oetzel, 2004). However, obtaining blood via venous puncture can be considered an animal welfare issue in public perception (Solano et al., 2004). Further, blood collection at multiple time points may be required for metabolic assessment, highlighted by Hussein et al. (2020), who recently reported diurnal variations in serum metabolites concentrations, such as glucose, β-hydroxybutyric acid, and urea from dairy cows. Therefore, diagnostic capacity using non-invasively obtained biological fluid such as milk could prove to be a useful alternative to blood in determining cow metabolic and health status. Previously, Zhao et al. (2016) reported that different levels of dietary NDF and starch altered milk fat, protein, and lactose content and production. In another study, milk protein content increased when cows were fed diets rich in starch, partly due to an increase in the rumen’s available energy (Cantalapiedra-Hijar et al., 2014). Puppel et al. (2021) recently reported that the concentrations of selected milk whey proteins were altered due to ketosis and suggested whey proteins could be used as biomarkers for diagnosing biological dysfunction and metabolic diseases. Evaluating potential noninvasive biomarkers for diagnosing diet-induced rumen dysfunction may be used to diagnose metabolic shifts resulting from high-RFS and low-NDF diets. We hypothesized that the milk proteome would change in response to a low-peuNDF240, high-RFS diet. More specifically, we hypothesized that the abundance of immune-associated proteins in milk would change due to a high-RFS diet. This study aims to characterize the milk proteome in response to feeding cows a low-peuNDF240, high-RFS diet and relate this milk proteome to the characterized rumen metaproteome.

## MATERIALS AND METHODS

### Animals and Experimental Design

The experimental protocol for this study was approved by the William H. Miner Agricultural Research Institute Animal Care and Use Committee (IACUC# 2017AUR02). Samples analyzed in this experiment were collected from a larger study conducted at the William H. Miner Agricultural Research Institute (Chazy, NY) from July 2019 to September 2019. The experimental design of the larger study used 8 lactating ruminally cannulated Holstein cows (85 ± 15 DIM) included in a 4 × 4 Latin square experiment with a 2 × 2 factorial treatment design consisting of 4 28-d periods as described by Smith (2021). The 8 cows used in this study were cannulated at the beginning of their previous dry (nonlactating) period, and these cannulas were cleaned regularly throughout the dry period and lactation. The rumen cannulation was performed according to the William H. Miner Agricultural Research Institute guidelines. Briefly, the procedure was a one-step fistulation procedure whereby a circular incision was created in the left paralumbar fossa after analgesic and anesthetic administration, subsequently followed by an incision in the rumen wall. The rumen wall was sutured to the abdominal skin to form a fistula, into which a 3-inch flexible rubber cannula (Bar Diamond, Parma, ID) was placed. The surgery site was inspected until healed, and the vital signs of the cows were recorded throughout the inspection. The 3-inch cannula was replaced with a 4-inch cannula after d 14 or when well healed. In each period, cows were paired randomly and assigned to one of 4 dietary treatments (DM basis) of different concentrations of RFS and peuNDF240 content in the diet. Blinding was not used. For this study, samples were collected from the cows fed a diet containing low peuNDF240, high RFS (6.7% of DM, 19.2% of DM**; LNHR**) or high peuNDF240, low RFS (8.60% of DM, 16.7% of DM; **HNLR**) in each period. The complete diet ingredient and nutritional profiles can be found in the supplementary file (Supplemental Tables S1 and S2; https://data.mendeley.com/datasets/y6zkhkhnjg/1; Mulakala, 2023).

### Rumen Fluid Sampling and Protein Fractionation

Rumen fluid samples were collected from each cow in each period on d 26 (1400 h) and d 27 (0600 h and 1000 h), resulting in 42 samples [2 cows per treatment per period × 2 treatments per period × 4 periods × 3 samples per cow per period; 6 samples were not collected]. As described by Steele et al. (2012), rumen fluid samples were collected from 3 areas in the mid-ventral ruminal sac of each cow’s rumen and pooled within the cow within the sampling point to create one composite sample per cow per time point. The samples were flash-frozen using a dry-ice ethanol bath and stored at −80 °C until further processing.

Rumen fluid samples were processed as described by Honan and Greenwood (2020). Frozen rumen fluid samples (n = 42) were thawed overnight at 4 °C and strained through 4 layers of cheesecloth (Lion Services Inc., Charlotte, NC) to separate coarse fibers. A universal control (**UC**) was generated by pooling equal volumes of rumen fluid from each cow at each time point across the 4 periods. The samples and UC were centrifuged at 16,000 × *g* for 20 min at 4 °C, and the supernatant was discarded to collect the cell pellets. One mini-tablet protease inhibitor (Pierce Protease Inhibitor Mini Tablets, Thermo Scientific, Rockford, IL) dissolved in RIPA lysis buffer, and a 5 mm stainless steel bead (Qiagen, Hilden, Germany) was added to tubes containing pellets. Sample pellets were homogenized (TissueLyser II, Qiagen) at 30 Hz for 30 s, followed by a 3-min incubation on ice. Six repetitions of this homogenization and incubation process were performed. The resulting homogenate samples were precipitated by adding a 6 M trichloroacetic acid/80 m*M* dithiothreitol solution at a 1:3 protein extract ratio, and then samples were incubated overnight at 4 °C. Following overnight incubation, samples were centrifuged at 16,000 × *g* for 20 min at 4 °C, and the supernatant was discarded. The collected pellets were washed twice with 20% dimethyl sulfoxide (**DMSO**) in acetone and twice with 100% ice-cold acetone. The wash step protocol was as follows: 20% DMSO in acetone was added to cell pellets, incubated for 1 h at −20°C, and then centrifuged at 10,000 × *g* for 5 min at 4°C to collect the cell pellet. The collected pellet was washed twice with 100% ice-cold acetone instead of 20% DMSO in acetone by following the wash step protocol described above. The collected pellets were air-dried and resuspended in phosphate-buffered saline and total protein extract concentrations were quantified using a bicinchoninic acid assay kit (Pierce, Rockford, IL).

### Milk Sample Collection and Milk Protein Isolation

Cows were milked thrice daily at 0430, 1230, and 2030 h throughout the study. Milk samples were collected from each cow on d 25 (2030 h), d 26 (0430 h, 1230 h and 2030 h), and d 27 (0430 h and 1230 h) of each period. Milk samples were collected using the commercially available in-line sampling unit attached to each cow’s milk line. At each milk sampling time point, a BouMatic (Madison, WI, USA) in-line sampling unit was attached to an individual milk meter. A subsample of the milk was automatically pumped into the sampling unit throughout the milking time for each cow, which provided a representative composite sample within cow within milking. The samples were thoroughly mixed in the sampling unit and then transferred into collection tubes. The collection tubes were flash-frozen using a dry-ice ethanol bath and stored at −80 °C until further processing.

After thawing milk samples (n = 94, including 6 milk samples per cow per period × 2 cows per treatment per period × 2 treatments per period × 4 periods; 2 samples were not collected) overnight at 4°C, the samples were composited within a period within cow within time point across the day, creating 47 samples that include 3 samples (one 0430 h sample, one 1230 h sample, and one 2030 h sample) per cow (n = 4) per period (n = 4). A UC was generated by pooling equal milk volumes from each cow at each time point across the 4 periods. A mammalian protease inhibitor cocktail (0.24 mL/g milk protein; Protease Inhibitor Cocktail, Sigma, Milwaukee, WI, USA) was added to each composite milk sample and the UC. Samples were then centrifuged at 4,000 × *g* for 15 min at 4°C. The cream layer was then removed, and the depletion of casein from skimmed samples was performed as described by Tacoma et al. (2016). Briefly, CaCl_2_ (60 m*M*) was added to the skimmed sample, and the pH was adjusted to 4.3 using 30% acetic acid (Fisher Scientific, Fair Lawn, NJ, USA). Samples were then ultra-centrifuged at 189,000 × *g* at 4°C for 70 min, and the supernatant was collected and stored at −80°C until processing. The samples were analyzed using the bicinchoninic acid assay kit (Pierce, Rockford, IL, USA) for protein quantification.

### TMT Peptide Labeling

Tandem mass tag (**TMT**) peptide labeling was performed using 10 plex TMT reagents kits (Thermo Scientific) per the manufacturer’s instructions. Briefly, 72 μg of protein from each rumen fluid and milk sample was aliquoted, and the volume was adjusted to 100 μL with 100 m*M* triethylammonium bicarbonate. Samples were incubated with 0.5 m*M* of dithiothreitol for 60 min at 37 °C and then with iodoacetamide for 30 min in the dark. After incubation, trypsin was added, and samples were digested overnight at 37 °C. The TMT reagents (0.8 mg dissolved in 41 μL of acetonitrile) were added to the digested samples and incubated for 1 h at room temperature. After incubation, 8 μL of 5% hydroxylamine were added to quench the reaction. Each multiplex was created by pooling an equal volume (25 μL) from each subset of individual TMT-labeled samples (9 experimental samples + 1 aliquot of UC per 10 plex TMT reagents kit). Rumen fluid (n = 42) and milk samples (n = 47) were processed separately. A 100 μL aliquot from each multiplex was dried to remove triethylammonium bicarbonate and then fractionated using a high-pH reversed-phase peptide fractionation kit (Thermo Scientific) as per kit instructions, resulting in 8 fractions for each multiplex. The fractionated samples were submitted to the Vermont Biomedical Research Network Proteomics Facility (The University of Vermont, Burlington, VT) for liquid chromatography-tandem mass spectrometry (**LC-MS/MS**).

### LC-MS/MS Analysis

The fractionated peptide samples were dissolved in 10 μL of 2.5% formic acid (**FA**), and 2.5% acetonitrile (**ACH**) in water for subsequent LC-MS/MS peptide identification and quantification. The LC-MS/MS analysis was performed on a Q-Exactive Plus MS coupled to an EASY-nLC 1200 (Thermo Scientific), similar to the methods described by Honan and Greenwood (2020). Briefly, samples were loaded onto a 100 μm × 500 mm capillary column packed with Halo C18 (2.7 μm particle size, 90 nm pore size, Michrom Bioresources, CA, USA) at a flow rate of 300 nL/min. Following solvent gradient conditions were used to separate peptides; 2.5 to 35% of ACH/0.1% FA for 60 min and increase from 35 to 100% ACH /0.1% FA in 1 min and then 100% ACH/0.1% FA for 4 min, an immediate return to 2.5% ACH/0.1% FA, and a final hold at 2.5% ACH/0.1% FA. Peptides were introduced into the MS via a nanospray ionization source, and laser pulled ~3 μm orifice with a spray voltage of 2.0 kV. Full MS scans were acquired in a data-dependent “Top 10” acquisition mode with lock mass function activated (*m*/*z* 371.1012). The scans were acquired in the mass range of 350–1,600 *m*/*z* with a mass resolution of 70,000, and the AGC target value was set at 1e^6^. The top 10 intense peaks in MS were fragmented with higher-energy collisional dissociation of 10. MS/MS spectra were obtained at a 35,000 resolution, with an AGC target of 1e^5^ and a maximum injection time of 100 ms. Dynamic exclusion was enabled (peptide match: preferred; exclude isotopes: on; underfill ratio: 1%).

### Data Analysis

Product ion spectra acquired from rumen fluid samples were searched using the SEQUEST search engine with percolator node (false discovery rate to less than 1%) on Proteome Discoverer 2.4 (Thermo Scientific) against 71 rumen-relevant composite databases downloaded on September 12, 2020. In contrast, product ion spectra acquired from milk samples were searched against the *Bos taurus* database downloaded from UniProt on October 28, 2021. Product ion spectra acquired from rumen fluid (n = 1) and milk samples (n = 3) from a cow with a high milk somatic cell count were excluded from the product ion spectra search and all further data analysis. Search Parameters were as follows: (1) full trypsin enzymatic activity; (2) maximum missed cleavages = 2; (3) minimum peptide length = 6, (4) mass tolerance at 10 mg/kg for precursor ions and 0.02 Da for fragment ions; (5) dynamic modifications on methionines (+15.9949 Da: oxidation), Dynamic TM-T6plex modification (The TMT6plex and TMT10plex have the same isobaric mass) on N-termini and lysines (229.163 Da); (6) static carbamidomethylation modification on cysteines (+57.021 Da). As Reporter Ions Quantifier node in the consensus workflow, the TMT-labeled peptides were quantified, and parameters were specified as follows: unique and razor peptides filter was set to the minimum of 1 for identification; Reject Quan results with missing channels and apply Quan value corrections were set as false; Co-Isolation Threshold value was set as 75; Average Reporter S/N Threshold was 10; “Total Peptide Amount” was used for normalization and Scaling Mode was set “on Control Averages,” so that the peptide abundances in the UC labels were set as 100 and the abundances in other channels were scaled accordingly. All the protein identification and quantification information (<1% FP; with protein grouping enabled) was exported from the MSF result files to Excel spreadsheets. The scaled abundance values of the samples were used for further statistical analysis. For any proteins identified as uncharacterized, the FASTA sequence was retrieved from UniProt (http://www.uniprot.org/) and searched through the Basic Local Alignment Search Tool (BLAST; http://blast.ncbi.nlm.nih.gov/Blast.cgi). The top hit protein was selected as an identified protein if the hit was 100% matched with the searched query. A mixed procedure model of SAS 9.4 (SAS Institute, Cary, NC) was performed, including diet, period, time, diet × time interaction as the fixed effects and cow, cow × diet × time as random effects. The diet and diet × time interaction effect on protein abundance was considered significant if *P* < 0.05. Further, the false discovery rate-adjusted *P*-value (***P*_FDR_**) was calculated to account for multiple comparisons using the MULTTEST procedure of SAS 9.4. The comparison was considered significant if the adjusted *P*_FDR_ < 0.05. Results of the mixed procedure are listed herein, and proteins that were identified as significant after the multiple comparisons procedure are specified.

### ELISA

An ELISA was used to validate the concentration of lipoprotein lipase (LPL) in milk samples. Although it was noted that the commercially available ELISA used in this study has not been extensively validated and has some limitations in terms of the linear range of detection, this assay is commercially available, allowing for repeatability and LPL detection within bovine milk. The LPL protein was selected for validation for 2 reasons 1) it is the protein that had the highest statistically significant affected by a diet and diet × time interaction and the effect of diet × time interaction on the LPL abundance remained significant after *P*-values were adjusted for multiple testing corrections, 2) LPL protein quantification with techniques such as ELISA using commercially available products is possible, whereas protein quantification assays or antibodies are not currently available for all differentially affected proteins. The concentration of LPL in the milk serum was measured in triplicate using a commercially available ELISA kit (Bovine Lipoprotein Lipase, MBS005972; My BioSource, Inc., San Diego, CA). The ELISA, including standard curve creation, was performed according to the manufacturer’s instructions. The kit’s sensitivity was 5 ng/mL, and the detection range of the kit was 25 to 800 ng/mL. Minimum acceptable R^2^ of the standard curve was 0.99. Both intra-assay CV (%) and interassay CV (%) of the kit were less than 15% [CV (%) = SD/mean × 100]. The data were analyzed using the same statistical model used to analyze the LC-MS/MS data as described above. The diet and diet × time interaction effect on protein abundance was considered significant if *P* < 0.05.

## RESULTS

### Rumen Metaproteome

The rumen meta-proteomic analysis in this study identified 129 proteins labeled in all samples across 24 searched microbes (Supplemental Table S3; https://data.mendeley.com/datasets/y6zkhkhnjg/1; Mulakala, 2023). Identified microbes and their strains were as follows: *Prevotella ruminicola*, *Prevotella ruminicola strain ATCC 19189*, *Ruminococcus albus 8*, *Ruminococcus albus strain 27210*, *Ruminococcus albus strain SY3*, *Ruminococcus bromii*, *Butyrivibrio hungatei*, *Prevotella aff. ruminicola Tc2–24*, *Methanobrevibacter ruminantium strain 35063*, *Eubacterium cellulosolvens 6*, *Ruminococcus flavefaciens*, *Ruminococcus flavefaciens 007c*, *Pseudobutyrivibrio ruminis*, *Selenomonas ruminantium*, *Eubacterium ruminantium*, *Pseudobacteroides cellulosolvens ATCC 35603*, *Clostridium aminophilum*, *Oxalobacter formigenes HOxBLS*, *Treponema saccharophilum DSM 2985*, *Butyrivibrio fibrisolvens*, *Butyrivibrio proteoclasticus strain ATCC 51982*, *Methanosarcina barkeri 3*, *Prevotella bryantii B14* and *Peptostreptococcus anaerobius CAG:621.* In the present study, 14 proteins across 9 microbial species were affected due to diet and diet × time interaction (Table 1). Of these 14 proteins, the abundance of 12 proteins across 8 microbial species was affected due to diet × time interaction, and only 2 proteins across 2 microbial species were affected due to diet. Among the 12 proteins affected by diet × time interactions, 6 proteins were affected due to diet at the 1400 h sampling point.

The abundances of pyruvate, phosphate dikinase (*Pseudo. ruminis*, *R. flavefaciens*) and NADH dehydrogenase (*R. albus SY3*) were higher in cows fed the HNLR diet compared with the cows fed the LNHR diet at 0600 h sampling point. At the same time point, the abundances of glutamate dehydrogenase (*Prevo. ruminicola*), fructose-bisphosphate aldolase (*Prevo. ruminicola*), and 60 kDa chaperonin (*Prevo. ruminicola strain ATCC 19189*) were higher in cows fed the LNHR diet compared with cows fed the HNLR diet. At the 1000 h sampling point, the abundances of glutamate dehydrogenase (*B. hungatei*), TonB-linked outer membrane protein, SusC/RagA family (*Prevo. ruminicola*), and pyruvate-ferredoxin/flavodoxin oxidoreductase (*P. bryantii B14*) were higher in cows fed the LNHR diet, whereas the abundance of NifU homolog involved in Fe-S cluster formation (*Pseudo. ruminis*) was higher in cows fed the HNLR diet. The abundance of NifU_N domain-containing protein (*P. cellulosolvens ATCC 35603*) and Glyceraldehyde-3-phosphate dehydrogenase (*P. bryantii B14*) was higher in the cows fed the HNLR diet compared with the cows fed with the LNHR diet at 1400 h sampling point.

The abundance of pyruvate, phosphate dikinase (*R. albus SY3*), and BMC domain-containing protein (*C. aminophilum*) were affected due to diet, and their abundance was higher in the cows fed the HNLR diet compared with those fed the LNHR diet. However, the effect of diet and diet × time interaction on rumen microbial protein abundances was not significant at a cutoff of *P*_FDR_ < 0.05, except for NifU homolog involved in Fe-S cluster formation abundance from *Pseudo. ruminis* at the 1000 h sampling point (Table 1).

### Milk Proteome Profile

Proteomic analysis identified 159 milk proteins labeled in all samples (Supplemental Table S4; https://data.mendeley.com/datasets/y6zkhkhnjg/1; Mulakala, 2023). Of these 159 identified proteins, the abundances of 21 proteins were affected due to diet and the diet × time interaction, including 19 proteins affected by the diet × time interaction (Table 2) and 2 proteins affected by diet. Among 19 proteins affected by the diet × time interaction, 16 were affected by the diet at the 0430 h sampling point, with 6 proteins having a higher abundance in milk collected from cows fed the LNHR diet compared with samples from those fed the HNLR diet.

The abundance of immunoglobulin M heavy chain secretory form, kininogen-1, inter-α-trypsin inhibitor heavy chain H4, complement factor H, monocyte differentiation antigen CD14, complement factor I isoform X1, ribonuclease 4, inter-α-trypsin inhibitor heavy chain H2 were lower in milk from LNHR-fed cows at 0430 h collection time point compared with samples from the HNLR-fed cows, whereas the abundances of α_S1_-casein, κ-casein, and secretoglobin family 1D member were higher in milk samples from LNHR-fed cows at the same collection time point. The abundances of mitogen-activated protein kinase 5, complement component C9 and vitamin D-binding protein were higher in milk from cows fed the HNLR diet at 1230 h collection time point compared with milk samples from those fed the LNHR diet.

The abundance of CTSL1 and lipoprotein lipase (LPL) were affected due to diet. The abundance of the CTSL1 protein was higher in cows fed the HNLR diet (112 vs. 90; *P* = 0.03), whereas the abundance of LPL was higher in cows fed the LNHR diet (1210 vs. 604; *P* = 0.01). However, the effect of diet and diet × time interaction on milk protein abundances was not significant at a cutoff of *P*_FDR_ < 0.05, except for LPL abundance at 0430 h sampling point. The abundance of LPL was higher in milk from LNHR-fed cows at 0430 h collection time point compared with samples from the HNLR-fed cows (*P*_FDR_ = 0.01; Table 2)

### Lipoprotein Lipase ELISA Validation

**Figure 1.**
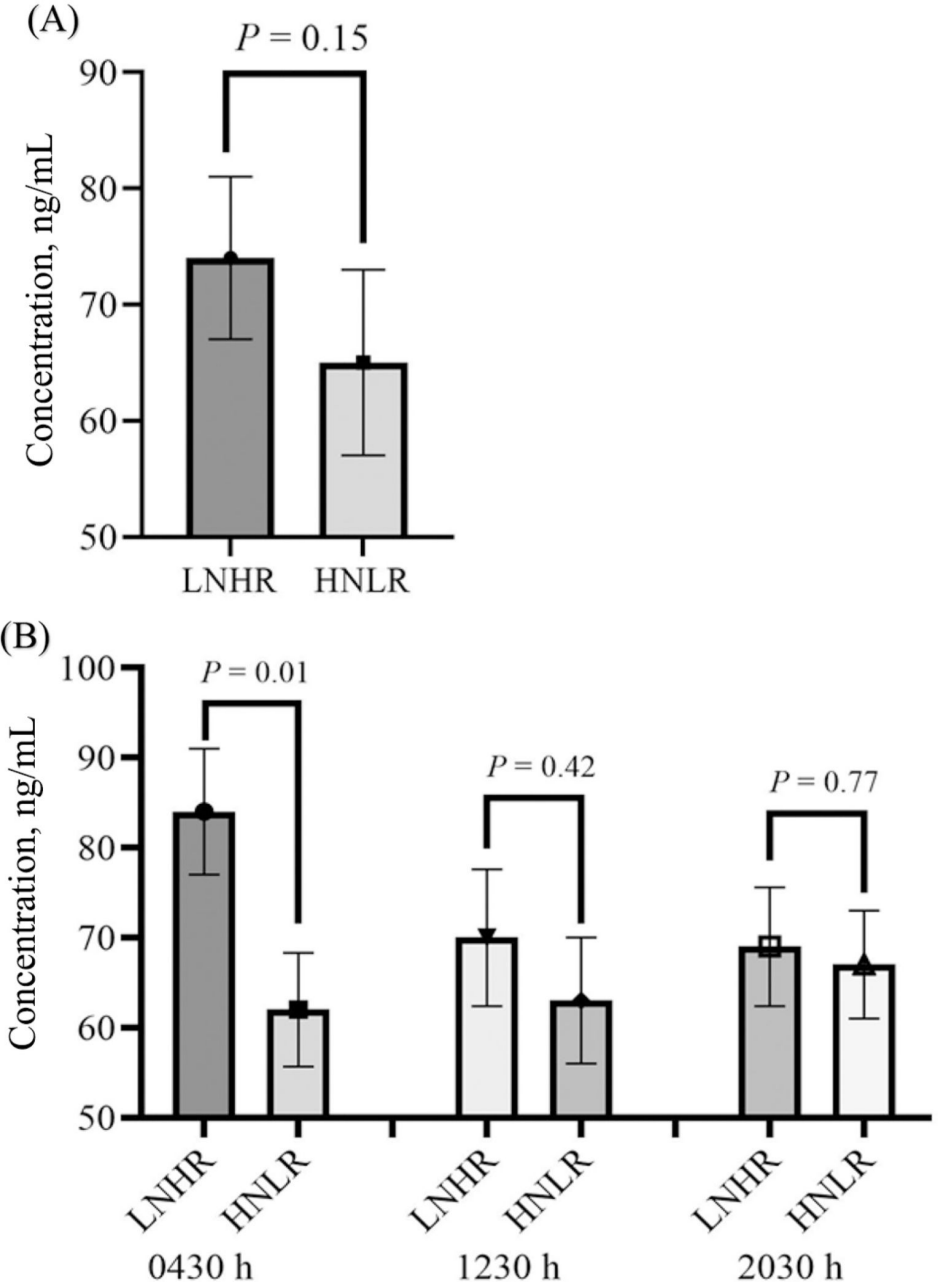
(A) Average lipoprotein lipase concentration in the milk from cows fed diets with low physically effective undegraded NDF at 240 h and high rumen-fermentable starch content (LNHR), or high physically effective undegraded NDF at 240 h and low rumen-fermentable starch content (HNLR), determined by ELISA. (B) Average lipoprotein lipase concentration in the milk from cows fed with the LNHR or HNLR diets at different sampling time points, determined by ELISA. Mean (± SE) milk lipoprotein lipase concentrations are reported in ng/mL. n = 8 cows per treatment (LNHR or HNLR) per time point.

The concentration of LPL, as determined by ELISA, was higher in milk samples from cows fed the LNHR diet compared with milk samples from cows fed the HNLR diet at the 0430 h sampling point (*P* = 0.01; Figure 1B). This treatment difference was not observed for the other milk sampling times (*P* = 0.42 and 0.77 for sampling at 1230 and 2030 h, respectively; Figure 1B). The concentration of LPL was not significantly affected by diet (*P* = 0.12; Figure 1A) when the time was not considered.

## DISCUSSION

Previous research clearly demonstrates that the diet’s concentration and profile of fiber and starch can influence milk composition and component profiles produced by dairy cows. For example, AlZahal et al. (2009) observed that cows fed a lower-fiber diet (32.7% of DM) produced lower levels of polyunsaturated fatty acids in milk compared with cows fed with a higher-fiber diet (40% of DM). Zhao et al. (2016) reported a linear decrease in milk protein and lactose content as the diet’s NDF:starch ratio increased. While the higher NDF:starch results in decreased propionate availability for gluconeogenesis and consequently less lactose synthesis occurs as a result, the effect of these diets on glycolysis is more complex. In the larger study wherein the samples for the current trial were collected, Smith (2021) outlined that both the lactose content (4.57 vs. 4.59%) and yield (2.38 vs. 2.35 kg/d) were similar across the LNHR versus HNLR treatments, respectively. This is indicative that the higher starch intake by the LNHR treatment (0.03 kg/d more than the HNLR treatment group) likely supported additional glucose needs of the immune system. Indeed, in the current trial, the LNHR diet affected the abundance of 21 milk proteins isolated from the skim milk fraction, and most of the milk proteins affected by the LNHR diet in this study were immune-related, suggesting systemic inflammation. As cows were cannulated at the beginning of their dry period, and the study was not started until 80 d DIM, it indicates that systemic inflammation was probably not due to rumen cannulation during the study. Previous studies evaluating the effect of subacute ruminal acidosis (**SARA**), typically resulting from highly fermentable and low-fiber diets, may provide insight into this observed protein response. In the larger study associated with this trial, Smith (2021) reported that cows fed the LNHR diet had a rumen pH <5.8 for approximately 5.8 h/d. According to Zebeli et al. (2008), cows with a rumen pH lower than 5.8 for more than 5 to 6 h/d are at a high risk of developing SARA. Therefore, cows fed the LNHR diet in this study could be assumed to be at risk. Previous studies have reported that rumen pH depression increases free ruminal concentrations of LPS (Gozho et al., 2007; Emmanuel et al., 2008). One supported postulation is that rumen pH depression and increased free ruminal LPS may damage ruminal epithelium (Nagaraja and Titgemeyer, 2007; Plaizier et al., 2012; Aschenbach et al., 2019) and thereby increase the translocation of LPS from the gut into the blood circulation (Plaizier et al., 2012; Aschenbach et al., 2019). Translocation of LPS into the blood circulation provokes an inflammatory response (Guo et al., 2017). In response to diet-induced systemic inflammation, the mammary gland shifts its priorities from synthesizing milk components to repartitioning more nutrients toward synthesizing immune molecules (Dong et al., 2011). This homeorhetic reprioritization in response to diet was recently highlighted in the research of Ma et al. (2022), who reported that feeding a high-concentrate diet to dairy cows increased the blood LPS concentration within the lactic vein, increasing the abundance of proinflammatory cytokines (IL-6 and IL-1α), immune-associated factors (lingual antimicrobial peptide and tracheal antimicrobial peptide) in the mammary gland. Even though we did not measure blood or free ruminal LPS concentrations in the current trial, this previously characterized relationship could provide insight into our observations.

To confirm the dietary induction of altered rumen microbial function in the current study, we did observe shifts in the abundance of microbial proteins due to a diet × time interaction. In this current trial, the abundance of 12 proteins across 8 microbial species was affected due to the diet × time interaction, highlighting the relevance to diurnal variation in rumen metaproteome characterization studies. This aligns with our previous research, where we investigated the effect of not only LNHR and HNLR diets on the rumen metaproteome but also the effect of diets high in peuNDF240 and RFS (HNHR) and low in peuNDF240 and RFS (LNLR; Mulakala et al., 2022). In agreement with our previous observations, many of the affected proteins were associated with glycolysis pathway in terms of functionality. For example, the abundance of pyruvate phosphate dikinase from *Pseudo. ruminis* and *R. flavefaciens*, which is involved in glycolysis and gluconeogenesis pathways, was higher in the cows fed the HNLR diet at the 1400 h sampling point in the current study. At the same time, the abundance of fructose-bisphosphate aldolase from *Prevo. ruminicola*, which is also involved in the glycolysis and gluconeogenesis pathways, was higher in the cows fed with the LNHR diet.

The results of the rumen metaproteome in this study indicate an alteration of microbial metabolic activity due to the LNHR diet, which can subsequently alter the fermentation type and ultimately affect the composition of bodily fluids such as milk. For example, studies have shown that; milk fat was decreased because of the changes in the concentration of volatile fatty acids and the alteration of microbiota in the rumen due to the high-starch diet in dairy cows (Pitta et al., 2018; Zeng et al., 2019). Therefore, in this current experiment, we sought to identify milk proteins that could be potential biomarkers of a diet-induced altered ruminal environment. Identifying potential biomarkers of diet-induced altered ruminal environment can be rapid, pertinent decision tools as they provide snapshots of cow metabolic status and help evaluate the effect of diet on rumen health. Herein we successfully identified 21 milk proteins affected by the peuNDF240 and RFS content of the diet, including several milk protein candidates that may be appropriate biomarkers indicative of an altered rumen environment. The diet affected the abundance of 16 milk proteins, with the 0430 h sampling point specifically being distinctive. In addition, most of the proteins affected by diet were immune-related. These findings cumulatively suggest that biological shifts resulting from diet-induced rumen changes can be reflected in milk but that these changes are not diurnally uniform across milking times. To identify the most representative biomarker with a feasible prospect of industry application, both aspects require consideration.

In terms of biological assessment, we observed a lower abundance of RNase4 in milk from cows fed the LNHR diet. Several proteins within the ribonuclease (RNase) family are present in cow milk and have previously been reported to have host defense-associated activities. For example, RNase family proteins, such as eosinophil cationic protein and eosinophil-derived neurotoxin, have been reported to have antibacterial and antiviral properties, respectively (Lehrer et al., 1989; Rosenberg and Domachowske, 2001). Previously, RNase4 in bovine milk has been reported to have antimicrobial activity against *Candida albicans* (Harris et al., 2010). Further, Murata et al. (2013) reported that the antimicrobial activity of lactoferrin and lactofericin was enhanced in the presence of RNase4, suggesting that lactoferrin, lactofericin and RNase4 may be concomitantly active antimicrobial agents in milk. The lower measured abundance of milk RNase4 in the current study may suggest immune suppression in the LNHR-fed cows. To support this possible explanation, we also observed a decreased abundance of immunoglobulin M heavy chain secretory form, and immunoglobulin-like protein in milk from cows fed the LNHR diet. This supports further investigation of these proteins as biomarkers of immune function but would not likely serve as specific biomarkers indicative of shifts in rumen function resulting from dietary carbohydrate profiles.

The CD14 protein is a glycosylphosphatidylinositol-anchored protein expressed on the membrane of macrophages and neutrophils (Haziot et al., 1988; Wright et al., 1991), It also serves as an immune cellular receptor for interactions with LPS. In this study, we observed lower abundance of CD14 in milk samples from LNHR-fed cows compared with samples from those fed the HNLR diet. The primary source of soluble CD14 (**sCD4**) in body fluids is from shedding of membrane-bound CD14 from monocytes and neutrophils (Bazil and Strominger, 1991; Haziot et al., 1993). Studies have shown that sCD14 modulates immune responses by interacting with B and T lymphocytes (Arias et al., 2000; Filipp et al., 2001) and plays a measurable role in supporting host defense in response to LPS challenges. For example, recombinant bovine sCD14 decreased the mortality of mice during an intraperitoneal LPS challenge (Lee et al., 2003b). In another study, the administration of recombinant sCD14 prevented LPS-induced TNF-α production in whole human blood (Haziot et al., 1994). Previously, it has been reported that SARA stimulated the expression of genes associated with the functions of LPS receptors, such as CD14 in white blood cells (Stefanska et al., 2018). Although sCD14 has been primarily studied in blood, identifying this protein in milk is not new. Lee et al. (2003a) reported that milk sCD14 abundances were increased with intramammary LPS challenge in cows and speculated that possible sources of sCD14 in milk could result from serum leakage or cells in the mammary gland. However, Lee et al. (2003a) also observed that milk collected from the mastitis-infected quarters had lower sCD14 than uninfected quarters of the same cow; the possible explanation outlined by Lee et al. (2003a) was that increased sCD14 resulted in an increase in binding to the bacteria that could not be detected in the whey samples due to their removal by centrifugation. Previously, the direct binding of sCD14 to gram-negative bacteria has been reported (Jack et al., 1995). Ultimately, it could be postulated that the decrease in the abundance of milk sCD14 protein in samples from cows fed the LNHR diet in the current trial may be because either (1) it is less detected due to increased sCD14 association with LPS and their removal by centrifugation, or (2) decrease in shedding of sCD14 by neutrophils and macrophages into the milk. Even though this protein warrants further exploration, it is not likely the optimal biomarker for targeted identification of dietary carbohydrate-induced shifts.

The acute-phase immunological response elevates acute-phase proteins in animal circulation and tissues because of infection, inflammation, or trauma. Acute-phase proteins, such as serum amyloid A, and haptoglobin, have been evaluated as potential biomarkers of mastitis in cattle milk (Grönlund et al., 2003; Miglio et al., 2013). Recently, studies have focused on identifying new acute-phase protein inter-α-trypsin inhibitor heavy chain 4 (**ITIH4**) in the milk and whey of cow milk during mastitis (Huang et al., 2014; Soler et al., 2019). Boehmer (2011) suggested ITIH4 and kininogen can be potential candidates for inflammatory biomarkers in bovine milk. Previously, studies have shown the increase of ITIH4 in different inflammatory processes (Piñeiro et al., 1999; Jostins et al., 2012). However, an essential indicator of the disease process decreases the ITIH4 level. For example, the ITIH4 protein was not detected in the blood serum of patients with acute ischemic stroke compared with the control group; the serum levels of ITIH4 returned to normal as acute ischemic stroke patients recovered (Kashyap et al., 2009). Similarly, the lower abundance of ITIH4 in milk from cows fed the LNHR diet may indicate a diet-induced inflammatory response. Further decreases in the abundance of ITIH4 suggest immune suppression in cows fed the LNHR diet, as the role of the acute-phase response is to restore a healthy physiological function (Kushner, 1982). Additionally, kininogens are precursor proteins for kinins and are known to play key roles in complement activation (Discipio, 1982). Similar to ITIH4, the abundance of kininogen-1 was lower in milk from cows fed the LNHR diet, indicating immune suppression. To support these suggestions, we observed a decrease in the abundance of complement factor I isoform X1, complement factor I isoform X1, and inter-α-trypsin inhibitor heavy chain H2 in milk from cows fed the LNHR diet. Although each of these proteins may be potentially valid biomarkers, not all of these proteins can currently be analyzed via commercially available kits, reducing the rapidity of application.

Mitogen-activated protein kinase (MAPK) families are involved in various cellular processes, such as cell proliferation and differentiation, transcriptional regulation, and development. Mitogen-activated protein kinase kinase 5 (**MKK5**) is involved in the innate immune MAPK signaling cascade. Moyes et al. (2010) reported that MAPK9 is implicated as part of cows’ immune response to intramammary challenges and negative energy balance. Memon et al. (2019) recently reported that feeding a high-concentrate diet to lactating dairy cows alters the udder activity by triggering the MAPK-Nrf2 signaling pathway. In this current trial, we did not measure the LPS concentration, which may have explained our observation of an increase in the abundance of MKK5 in milk from LNHR-fed cows. However, an increase in the abundance of MKK5 may suggest MAPK signaling pathway was triggered in the cows fed with the LNHR diet. Therefore, further exploration of the effect of diet on the MAPK signaling pathway proteins in the mammary gland could support further characterization and identification of potentially valid biomarkers.

Lipoprotein lipase (**LPL**) was a protein of focus herein, as the abundance of lipoprotein lipase LPL in milk was affected by diet and diet × time interaction in this current study. Lipoprotein lipase is an enzyme associated with triacylglycerol hydrolysis in chylomicrons and very low density lipoproteins, converting each triacylglycerol into glycerol and nonesterified fatty acids for tissue utilization (Stins et al., 1992; Shingfield et al., 2010). The mammary gland takes up long-chain fatty acids through the action of LPL. The transcript abundance and activity of LPL markedly increase at the onset of lactation to augment the availability of fatty acids to the mammary tissue (Shirley et al., 1973). Alternatively, the transcription of LPL in the mammary gland is decreased by feeding a high-concentrate diet in dairy, goats, and cows (Zhao et al., 2014; Ma et al., 2022). Surprisingly, the abundance of LPL was higher in milk from cows fed the LNHR diet in the current trial. We performed an ELISA on the same samples to validate the higher LPL identified by LC-MS/MS. Though a lower magnitude of change, we similarly measured higher concentrations of LPL in milk from cows fed the LNHR diet. As measured through the ELISA validation, the concentration of LPL was significantly higher in the milk from cows fed the LNHR diet at the 0430 h sampling point, supporting our suggestion that biological shifts reflected in the milk due to diet-induced rumen changes are not diurnally uniform across milkings. Studies have reported that LPL is primarily regulated at transcription and post-transcription levels (Doolittle et al., 1990; Tengku-Muhammad et al., 1998; Hughes et al., 2002). Therefore, *LPL* mRNA expression is not always accompanied by corresponding increases in LPL synthesis. For example, when rat adipocytes were treated with epinephrine, we observed a decrease in LPL synthesis with no corresponding change in *LPL* mRNA level (Ong et al., 1992). Jensen et al. (1994) reported that LPL is also regulated at transcription and post-transcriptional levels in the mammary gland of the mouse. Increased abundance of LPL in milk from cows fed the LNHR diet may result from differentially regulating LPL at transcription and post-transcriptional levels. The results of this study indicate that LPL can be a potential biomarker candidate for diet-induced rumen microbial changes and should be further investigated.

## CONCLUSIONS

In the present study, 14 rumen microbial proteins across 9 microbial species were affected due to different diet combinations of peuNDF240 and RFS, with the abundances of 12 microbial proteins across 8 microbial species being affected due to the diet × time interaction. Concurrently, 21 milk proteins were affected by dietary treatment, with the abundance of 19 milk proteins being affected due to the diet × time interaction. The higher concentration of LPL in milk from cows fed the LNHR diet, identified by LC-MS/MS analysis and validated with ELISA, suggests that biological shifts resulting from diet-induced changes to the rumen microbial environment can be reflected in milk, and milk proteins may be feasible biomarkers for such changes with careful consideration for function and diurnal variability.

## Figures and Tables

**Table 1.** Effects of diet with low physically effective undegraded NDF at 240 h (peuNDF240) and high rumen-fermentable starch (RFS), fed to lactating Holstein dairy cows, on rumen microbial protein abundances at different sampling time points^1^

			0600 h		1000 h		1400 h			
Accession no.	Protein	Species	HNLR^2^	LNHR^3^	HNLR	LNHR	HNLR	LNHR	*P*-value	*P*_FDR_^4^

A0A1H7FBX7	Pyruvate, phosphate dikinase	*Pseudobutyrivibrio ruminis*	84^a^	88^a^	91^a^	85^a^	98^a^	82^b^	<0.05	0.28
A0A1M6YX51	Glutamate dehydrogenase	*Prevotella ruminicola*	101^a^	111^a^	102^a^	104^a^	93^a^	124^b^	<0.05	0.37
A0A1M7L8N4	Pyruvate, phosphate dikinase	*Ruminococcus flavefaciens*	91^a^	92^a^	105^a^	106^a^	112^a^	95^b^	<0.05	0.23
D5EWS5	60 kDa chaperonin	*Prevotella ruminicola (strain ATCC 19189)*	125^a^	129^a^	112^a^	94^a^	89^a^	123^b^	<0.05	0.26
A0A1M6VCT7	Fructose-bisphosphate aldolase	*Prevotella ruminicola*	136^a^	124^a^	117^a^	109^a^	101^a^	146^b^	<0.05	0.26
A0A011VU56	NADH dehydrogenase	*Ruminococcus albus SY3*	98^a^	107^a^	125^a^	114^a^	116^a^	84^b^	<0.05	0.28
A0A1D9P306	Glutamate dehydrogenase	*Butyrivibrio hungatei*	93^a^	98^a^	91^a^	106^b^	92^a^	99^a^	<0.05	0.29
A0A1H7G5V3	NifU homolog involved in Fe-S cluster formation	*Pseudobutyrivibrio ruminis*	105^a^	83^a^	124^a^	80^b^	90^a^	84^a^	<0.05	0.04
D8DWJ0	Pyruvate-ferredoxin/flavodoxin oxidoreductase	*Prevotella bryantii B14*	107^a^	109^a^	105^a^	126^b^	98^a^	99^a^	<0.05	0.40
A0A1H6LFS4	TonB-linked outer membrane protein, SusC/RagA family	*Prevotella ruminicola*	97^a^	96^a^	91^a^	116^b^	111^a^	112^a^	<0.05	0.27
A0A0L6JXI6	NifU_N domain-containing protein	*Pseudobacteroides cellulosolvens ATCC*	69^a^	55^b^	76^a^	68^a^	78^a^	73^a^	<0.05	0.25
D8DZJ1	Glyceraldehyde-3-phosphate dehydrogenase	*Prevotella bryantii B14*	131^a^	108^b^	126^a^	122^a^	113^a^	123^a^	<0.05	0.36

a Mean values in the sampling point with different superscripts differ (*P* < 0.05).

b Mean values in the sampling point with different superscripts differ (*P* < 0.05).

1 n = 8 cows per treatment (LNHR or HNLR) per time point.

2 HNLR = high-peuNDF240 and low-RFS diet.

3 LNHR = low-peuNDF240 and high-RFS diet.

4 *P*_FDR_ = false discovery rate-adjusted *P*-value.

**Table 2.** Effects of a diet with low physically effective undegraded NDF at 240 h (peuNDF240) and high rumen-fermentable starch (RFS), fed to lactating Holstein cows, on milk protein abundances at different milking times^1^

		0430 h		1230 h		2030 h			
Accession no.	Protein	HNLR^2^	LNHR^3^	HNLR	LNHR	HNLR	LNHR	*P*-value	*P*_FDR_^4^

A0A140T8A9	κ-Casein	260^a^	479^b^	303^a^	276^a^	479^a^	410^a^	<0.05	0.24
A0JNP2	Secretoglobin family 1D member	144^a^	357^b^	229^a^	256^a^	357^a^	304^a^	<0.05	0.42
E1BI01	Mitogen-activated protein kinase 5	64^a^	66^a^	53^a^	118^b^	66^a^	92^a^	<0.05	0.44
F1MMD7	Inter-α-trypsin inhibitor heavy chain H4	104^a^	70^b^	89^a^	85^a^	70^a^	88^a^	<0.05	0.42
F1MNT4	Laminin subunit β 1	90^a^	56^b^	72^a^	66^a^	56^a^	73^a^	<0.05	0.25
F1MNV5	Kininogen-1	98^a^	71^b^	84^a^	90^a^	71^a^	90^a^	<0.05	0.28
F1MNW4	Inter-α-trypsin inhibitor heavy chain H2	105^a^	77^b^	97^a^	104^a^	77^a^	92^a^	<0.05	0.45
F1N4M7	Complement factor I isoform X1	87^a^	63^b^	74^a^	85^a^	63^a^	77^a^	<0.05	0.41
F1N5M2	Vitamin D-binding protein	80^a^	64^a^	68^a^	86^b^	64^a^	75^a^	<0.05	0.43
G5E5T5	Immunogloin M heavy chain secretory form	81^a^	62^b^	81^a^	75^a^	62^a^	68^a^	<0.05	0.40
P02662	α_S1_-Casein	256^a^	562^b^	430^a^	405^a^	562^a^	462^a^	<0.05	0.14
P11151	Lipoprotein lipase	428^a^	1331^b^	720^a^	1042^a^	666^a^	1256^b^	<0.05	<0.01*
P17690	β-2-Glycoprotein	89^a^	56^b^	82^a^	92^a^	56^a^	91^a^	<0.05	0.42
P79345	Epididymal secretory protein E1	111^a^	73^b^	83^a^	95^a^	73^a^	97^a^	<0.05	0.47
Q0IIH5	Nucleobindin 2	86^a^	64^b^	77^a^	87^a^	64^a^	86^a^	<0.05	0.43
Q28085	Complement factor H	173^a^	134^b^	177^a^	138^a^	134^a^	162^a^	<0.05	0.39
Q3MHN2	Complement component C9	70^a^	69^a^	62^a^	86^b^	69^a^	90^a^	<0.05	0.43
Q3T000	Synaptobrevin homolog YKT6	100^a^	77^b^	95^a^	89^a^	77^a^	103^a^	<0.05	0.45
Q58DP6	Ribonuclease 4	98^a^	67^b^	80^a^	93^a^	67^a^	94^a^	<0.05	0.45
Q95122	Monocyte differentiation antigen CD14	106^a^	76^b^	92^a^	85^a^	76^a^	92^a^	<0.05	0.17

a Mean values in the sampling point with different superscripts differ ( *P* < 0.05).

b Mean values in the sampling point with different superscripts differ ( *P* < 0.05).

1 n = 8 cows per treatment (LNHR or HNLR) per time point.

2 HNLR = high-peuNDF240 and low-RFS diet.

3 LNHR = low-peuNDF240 and high-RFS diet.

4 *P*_FDR_ = false discovery rate-adjusted *P*-value.

* *P*_FDR_ value representing the 0430 h sampling point.
